# Supplementary material for: MG-DIFF: A novel molecular graph diffusion model for molecular generation and optimization
Source: PLoS One. 2025 Oct 16;20(10):e0331450. doi: 10.1371/journal.pone.0331450 (PMC12530617; doi:10.1371/journal.pone.0331450)
Supplement: S1 Text — Includes detailed descriptions of the MG-DIFF model architecture, training hyperparameters, and definitions of evaluation metrics used in molecular generation and optimization tasks. (DOCX) [file pone.0331450.s001.docx]

**Supplementary**

**Model Configuration:** our graph transformer consists of 6 layers, each with eight attention heads, and a hidden size of 256. The model was trained for 500 epochs with a batch size of 128. We use the Adam optimizer and the learning rate is set as 1e-4, the maximum norm of the gradient is clipped to 1 to keep the stability of training. MG-DIFF requires moderate training time (e.g., ~20 hours on a single RTX 4090 for ZINC-250K), which is slightly more than VAE-based models but comparable to DiGress. The training benefits from efficient parallelization and stable convergence thanks to our mask-and-replace strategy.

**The evaluation metrics**

- Validity: the percentage of valid molecules a model is able to generate. If RDKit[41] was able to process the generated molecule object, then that molecule is valid.
- Novelty: the proportion of generated molecules that are not present in the training data.
- Diversity: the percentage of distinct molecules that the model is capable of generating.
- Internal Diversity(IntDiv*_p_*): measures the diversity of the generated molecules, which is a metric specially designed to check for mode collapse where the model repeatedly generates quite similar structures. This uses the power mean (p) of the Tanimoto similarity (T) between the fingerprints of all pairs of molecules in the generated set (G). We report IntDiv_1_ (p=1) and I IntDiv_2_ (p=2) in this work.

- Quantitative Estimation of Drug-Likeness (QED): is the geometric mean of eight common molecule properties(molecular weight, AlogP, hydrogen bond donors, hydrogen donor acceptors, molecular polar surface area, rotatable bonds, aromatic rings, structural alerts) and can estimate how well the molecule behaves in the human body, This value can be range from 0 to 1, and the higher the QED value, the similar a molecule is to existing drugs and will have higher chance surviving the drug-discovery process
- Synthetic Accessibility Score (SAS): is an estimation of the ease of synthesizing a molecule, determined by calculating a fragment score that is adjusted based on the complexity of the molecule. The SAS value ranges from 0 to 10, with molecules scoring above 6 considered challenging to synthesize.

**Denoising network architecture**

The denoising network takes a noisy graph $\text{G}_{\text{t}}\text{=(}\mathbf{A}\text{,} \mathbf{C}\text{,} \mathbf{E}\text{)}$ as input and outputs tensors $\mathbf{A}\text{,} \mathbf{C} \text{and} \mathbf{E}$which represent the predicted distribution over clean graphs. To achieve this, we adopt a modified version of the graph transformer model. The proposed model comprises three main components: an embedding layer, several self-attention layers, and an output layer. Initially, we concatenate the tensors **A**, **C,** and some noise $\mathbf{X}\in\text{R}^{N\times(d_{A}+d_{C}+d_{N})}$to form node features. These noises allow GNNs to preserve the permutation-invariance characteristic of regular GNNs and facilitate the effective learning and integration of both local and global graph features. Additionally, RNI has been shown to effectively alleviate the issue of over-smoothing, a critical problem that significantly impacts the performance of graph-based models. Then we embed **X** and **E** into fixed-dimensional vectors $\mathbf{X}_{0}\in\text{R}^{N\times d}$, $\mathbf{E}_{0}\in\text{R}^{N\times N\times d}$(*d* is the predefined hidden size). To incorporate time information and possibly conditional information, we embedded them into a continuous vector and added them to the node embedding. Within the graph transformer layer, the input node tensor is first updated using a multi-head self-attention sub-layer. Here, to update the node representation, the edge representation is taken into account during the calculation of attention.

$$\mathbf{Q}_{m-1}^{h}\text{=}\mathbf{Y}_{m\text{-}1}^{h}\mathbf{W}_{mQ}^{h},\mathbf{K}_{m-1}^{h}\text{=}\mathbf{Y}_{m\text{-}1}^{h}\mathbf{W}_{mK}^{h},\mathbf{V}_{m-1}^{h}\text{=}\mathbf{Y}_{m\text{-}1}^{h}\mathbf{W}_{mV}^{h},$$

$$\mathbf{A}_{m}^{h}\text{=}\frac{\mathbf{Q}_{m}^{h}\text{(}\mathbf{K}_{m}^{h})^{T}}{\sqrt{d_{K}}},\mathbf{O}_{m}^{h}=\mathrm{softmax}(\mathbf{A}_{m}^{h}+b_{m}^{h}(\mathbf{E}))\mathbf{V}_{m}^{h}$$

$$\mathbf{X}_{m}\text{=concat(}\mathbf{O}_{m}^{0}\text{,}\mathbf{O}_{m}^{1},...,\mathbf{O}_{m}^{H}\text{)}\mathbf{W}_{\text{O}}$$

where$\mathbf{Y}_{m\text{-}1}^{h}\in\text{R}^{n\times d/H}$ comes from evenly dividing the input $\mathbf{X}_{m-1}^{h}\in\text{R}^{n\times d/H}$ into *H* chunks; $\mathbf{W}_{mQ}^{h}\in\text{R}^{d/H\times d/H}, \mathbf{W}_{mK}^{h}\in\text{R}^{d/H\times d/H} \text{and} \mathbf{W}_{mV}^{h}\in\text{R}^{d/H\times d/H}$are learnable parameter matrices; *m* represents the m-th layer; *h* represents the h-th attention head and *H* is the total number of attention heads; $b_{m}^{h}$ is a fully connected neural network mapping the edge features to attention bias scalars.

After the self-attention sub-layer, the node feature and edge feature are both sent to an FFN sublayer. In this layer, the node information is injected to update the edge representation.

$$\mathbf{x}_{\text{i}}^{\text{m}}\text{=GELU(}\mathbf{o}_{i}^{m}\mathbf{W}_{m1}^{x}\text{)}\mathbf{W}_{m2}^{x}$$

$$\mathbf{e}_{ij}^{m}=\mathrm{GELU}(\mathrm{concat}(\mathbf{e}_{ij}^{m-1},\mathbf{x}_{i}^{m},\mathbf{x}_{j}^{m})\mathbf{W}_{m1}^{\text{e}})\mathbf{W}_{m2}^{\text{e}}$$

where $\mathbf{W}_{m1}^{x}, \mathbf{W}_{m1}^{x}, \mathbf{W}_{m1}^{x} and \mathbf{W}_{m1}^{x}\in R^{d\times d}$ are learnable parameter matrices.

Besides, all the self-attention sublayers and position-wise feed-forward sublayers are followed by a normalization layer and a residual connection to increase the generalization ability and utilize the original information.

The last layer includes three fully connected neural networks, which further process the Transformer layer's output and generate the predicted **A**, **C**, **E** tensors.

As can be seen, the proposed model is permutation invariant, allowing it to efficiently capture the essential features of graphs without the need for augmenting data with random permutations.
