# Supplementary material for: MG-DIFF: A novel molecular graph diffusion model for molecular generation and optimization
Source: PLoS One. 2025 Oct 16;20(10):e0331450. doi: 10.1371/journal.pone.0331450 (PMC12530617; doi:10.1371/journal.pone.0331450)
Supplement: Attachment — Submitted filename: 2025-plos-mgdiff.pdf [file pone.0331450.s002.pdf]

Review for MG-DIFF: A Novel Molecular Graph Diffusion Model for Molecular Generation and Optimization.

Summary: In this work, the authors present a new graph diffusion model specifically optimized towards molecular generation and optimization tasks. They propose three new strategies in model construction and training that improve model convergence (using mask-and-replace), capture local and global features using a graph transformer, and a graph padding strategy that enables the model to learn node distributions. While MG-DIFF achieves comparable performance on molecular generation benchmark on two datasets, further clarification is needed about the methodology and appropriate comparisons to prior work.

Major revisions:

1. The authors should provide additional theoretical justification for the choice of diffusion strategy, and show how well their mask-and-replace method improves their model performance. Comparative experiments explicitly isolating the contribution of the masking strategy would be ideal, but understandable if not included due to the training resources required (but should be mentioned then).
2. Similarly to above, ablation studies specifically demonstrating the contribution of the graph padding strategy towards conditional generation tasks would be useful.
3. It would be useful to include a discussion in the greater variance found in the SAS and QED metrics as there may be issues in obtaining consistent molecule quality in practical applications.
4. If possible, it would be good for the authors to include comparisons and a discussion of their method versus the DiGress method or other wider methods.
5. The authors discuss their molecular optimization approach, but do not discuss the practical limitations from a medicinal chemistry approach in that they cannot remove atoms.
6. It would be critical for the authors to clearly state the key details of the training process (e.g. hyperparameters, model starting conditions) to maximize reproducibility.
