# Supplementary material for: MG-DIFF: A novel molecular graph diffusion model for molecular generation and optimization
Source: PLoS One. 2025 Oct 16;20(10):e0331450. doi: 10.1371/journal.pone.0331450 (PMC12530617; doi:10.1371/journal.pone.0331450)
Supplement: Attachment — Submitted filename: responds.docx [file pone.0331450.s004.docx]

Response to Reviewers

We sincerely thank the reviewers for their constructive and insightful comments, which have significantly improved the quality of our manuscript. Below, we provide a point-by-point response to each comment. All changes made in the revised manuscript are marked in blue.

Reviewer Comments and Author Responses

Comment 1:

The authors should provide additional theoretical justification for the choice of diffusion strategy, and show how well their mask-and-replace method improves their model performance. Comparative experiments explicitly isolating the contribution of the masking strategy would be ideal...

Response:

Thank you for your insightful comment. We fully agree that the choice of noise corruption strategy is crucial in discrete diffusion models. In the revised manuscript, we have added a detailed theoretical motivation for the proposed mask-and-replace diffusion strategy in the Methods section (subsection: "Mask-and-replace diffusion strategy").

We emphasize that, compared to uniform corruption, the mask-and-replace approach:

Provides clear signal about corrupted locations, improving learning efficiency;Avoids severe semantic disruptions caused by replacing atom/bond types with uncorrelated categories;Enables the model to focus on recovering masked tokens while still allowing limited correction of unfit structures.Although we acknowledge that an ablation study would provide stronger empirical evidence, we were unable to include such an experiment due to limited computational resources. We have explicitly mentioned this limitation in the manuscript. Nonetheless, we highlight in the Results section that MG-DIFF achieves significantly better convergence and validity compared to existing models, which we attribute in part to the benefits of this targeted corruption strategy.

We hope the theoretical justification and observed empirical improvements provide sufficient support for the effectiveness of the mask-and-replace mechanism.

Comment 2:

Similarly to above, ablation studies specifically demonstrating the contribution of the graph padding strategy towards conditional generation tasks would be useful.

Response:

We appreciate the suggestion. Specifically, we note that while the padding strategy has a relatively limited impact on unconditional molecular generation performance (e.g., validity and novelty metrics remain largely unchanged when padding is removed), it is critical for enabling molecular optimization. Without a fixed-size representation across samples, our model would not be able to introduce additional atoms into the molecular graph in a principled manner. The padding nodes serve as placeholder positions that can be selectively activated during reverse diffusion, thus allowing for the addition of atomic groups required for property-driven molecule refinement.

We have made this distinction clear in the text and have emphasized that the main contribution of the padding strategy lies in providing the structural flexibility needed for local molecular optimization, not in boosting the performance of unconditional generation tasks.

Comment 3:

It would be useful to include a discussion in the greater variance found in the SAS and QED metrics as there may be issues in obtaining consistent molecule quality in practical applications.

Response:

Thank you for this valuable observation. We have expanded the Discussion section to address this issue which are marked red. We interpret the higher variance in SAS and QED as a reflection of our model’s ability to explore a wider chemical space, which is beneficial for early-stage drug discovery. At the same time, we acknowledge that such diversity may result in property inconsistency and suggest future strategies—such as property-aware training losses or post-generation filtering—to address this issue.

Comment 4:

Include comparisons and a discussion of their method versus the DiGress method or other wider methods.

Response:

We agree with this comment and have added a detailed discussion comparing MG-DIFF to DiGress in both the Introduction and Discussion sections which are marked red. We highlight that MG-DIFF addresses two key limitations of DiGress: (1) the use of a harsh uniform noise scheduler and (2) the inability to handle variable-size graphs. We also clarify that MG-DIFF supports conditional generation and optimization more flexibly than DiGress due to its mask-and-replace and graph padding strategies.

Comment 5:

The authors discuss their molecular optimization approach, but do not discuss the practical limitations from a medicinal chemistry approach in that they cannot remove atoms.

Response:

We appreciate this important remark. We have revised the Molecular Optimization and Discussion sections to explicitly acknowledge this limitation. Our current model is only capable of adding atoms and cannot delete atomic groups. We now discuss how this constraint affects optimization tasks such as SAS reduction and propose that future work could incorporate atom deletion through reversible masking or dynamic graph resizing techniques.

Comment 6:

It would be critical for the authors to clearly state the key details of the training process (e.g. hyperparameters, model starting conditions) to maximize reproducibility.

Response:

Thank you for the suggestion. We have added a new subsection titled Training Details in the Methods section, which provides comprehensive information about the optimizer, learning rate, batch size, network architecture, number of training epochs, hardware configuration, early stopping criteria, and initialization settings. This will ensure the reproducibility of our work.

Conclusion:

Once again, we sincerely thank the reviewer for the constructive feedback. We believe these revisions have significantly improved the clarity, depth, and reproducibility of our manuscript.
